# Supply Chain Partner Communication in a Managed Programme in the UK Water Industry: A Case Study with Social Network Analysis

**DOI:** 10.3390/ijerph16214211

**Published:** 2019-10-30

**Authors:** Qing Li, Shengqiao Wang, Nicky Shaw, Victor Shi

**Affiliations:** 1 Faculty of Management, Shanghai Business School, Shanghai 201400, China; liqing@sbs.edu.cn (Q.L.); wangsq@sbs.edu.cn (S.W.); 2 Department of Management, Leeds University Business School, Leeds LS2 9JT, UK; N.E.Shaw@lubs.leeds.ac.uk; 3 Lazaridis School of Business and Economics, Wilfrid Laurier University, Waterloo, ON N2L 3C5, Canada

**Keywords:** programme management, water supply chain, social network analysis, water sustainability

## Abstract

The water industry in every country aims to effectively and efficiently provide water with satisfactory quality in a sustainable and environmentally friendly manner. To this end, it is critical to achieve effective communication among the partners in water supply chain networks. In this paper, we focus on one of the UK’s largest water utility companies and its eight main contractors and analyze the factors influencing partner and network communication in a managed programme of their asset supply chain. We employ social network analysis to conduct the cross-sectional and longitudinal analysis of partner communication. Factors found to influence the communication network are grouping of projects within the programme, individual’s organisational affiliation, status, tenure, elapsed time through the programme lifecycle, and co-location. Our contributions to practice include demonstrating water programme management factors that influence communication and trust and how social network analysis can better inform them about intra- and interorganisational relationships. Moreover, the methodology introduced in this study may be applied to water management in other parts of the world.

## 1. Introduction

The privatisation of the water sector began in the UK in the late 1980s with the introduction of the 1989 Water Act. The conservative government at the time gave water companies monopolies in specific regions to provide services to residents and to businesses. Prior to privatisation, publicly owned geographically organised water boards were responsible for the water sector. Water privatisation established not only ten region-based geographically monopolized companies providing water and sewerage services but also regulatory organisations to oversee the industry and to ensure competition. Since then, the UK water industry has been under increasing pressure to provide clean water and to dispose of waste water in an efficient and environmentally sustainable manner [1] because of rising environmental standards, climate change, improving technology, adverse economic conditions, increasing customer expectations, and tightening of regulation (e.g., Ofwat, the key regulator, requesting radical innovations). Industry behaviour has been shaped by the regulator adopting five-year cycles for planning and implementing activity. In each five-year cycle, individual water companies undertake large investment programmes that agree with the regulator with the aim of improving the quality of services provided for customers and to advance efficiency and innovation within the water sector. The five-year cycle in accordance with the regulator’s five-year development framework sets the scene for the research context; to achieve resource sustainability and integration efficiency, the water company has to work in close partnership with contractors as their tier 1 suppliers. 

Prior research investigates how contractors or suppliers of complex projects collaborate and communicate for the provision of services as integrated solutions to the requirements of regulators in various sectors [2,3]. Scholars raise the question of a hybrid collaboration to coordinate and align performance across public–private partnerships (PPP) to achieve social value creation [4]. Nevertheless, UK PPP/Project financing (PFI) projects had employed standardised contractual forms, in part, reflecting increasing need to address communication network in other markets [2,5]. 

Contingency theory [6], an important theoretical lens in management research, asserts that environmental features such as industry sector and operation philosophy bear importantly on what are suitable structures and practices. Therefore, research on lesser-studied contexts and philosophies is needed to fill knowledge gaps for the benefit of both researchers and practitioners. Practitioners and researchers alike are interested in communication between supply chain partners because communication efficacy impacts markedly on supply chain performance through its contribution to supply chain integration (SCI) [7,8,9]. 

There are few empirical studies investigating the term “programme” or “programme management” as an approach to integrated management of multiple organisations and independent projects in, in particular, the water industry, where regulations are strong. In addition, extant Supply Chain Management (SCM) and SCI literature focuses mainly on the intraorganisational level of analysis, thus neglecting the importance of interorganisational relationships, important for communication within programme management. We remedy these gaps by studying how key factors affect communication and, hence, integration in the upper part of the supply chain for a five-year water programme of asset maintenance and renewal operated by a UK water company. Thus, the study addresses the following research question “How do key factors influence communication between partners in the top tiers of the supply chain of a UK water company’s asset management programme?” Data were collected longitudinally for months six and twelve of a five-year programme, and social network analysis was used to examine communication networks and how they evolve.

This paper contributes to both theory and practice. First, we contend that such a case study of SCI within a programme management ethos is novel. We believe the specific context for this programme management research is also novel given that the studied programme is a supply chain of construction projects in a privatized but monopolized utility sector. In this study, a UK water utility company (UC) delivers a programme of work to maintain and renew capital assets as agreed upon with the regulator; these programmes are called asset management plans (AMPs). As is typical of programme management [10], the time-limited programme comprises bundles of related projects where each project is be managed individually, but often, each bundle is also managed collectively as an integrated unit. Programmes in the water sector are distinguished from other programme types (e.g., financial programmes) by being comprised of thousands of related projects that differ in size and technical content. This volume and complexity require managerial structures to deal with project groupings. Communication within these structures is key to achieving integration and, hence, effectiveness.

Second, our study responds to this gap in the literature by studying key factors influencing communication between employees of the water company and its partners in the top tier of the supply chain of this novel programme. These include the company’s and individual’s affiliations to project groups, organisational affiliation of individual participants, physical proximity of participants, seniority and industry tenure of participants, and the elapsed time through the programme lifecycle. The study investigates how individuals affiliated with the various partnering organisations form new and extend existing collaborative relationships within the revised commercial and physical context of a new programme. It also addresses longitudinal relationship development in the programme. Using social network analysis (SNA) [11,12] as a main analytical method brings a further fresh aspect to programme management research. SNA permits analysis of communication at the individual level within both intra- and interorganisational supply networks.

The paper’s structure after this introduction is as follows. Relevant literature is reviewed and research propositions are presented in Section 2. In Section 3, the study’s methodology is presented with details, including those of the social network analysis (SNA) method. In Section 4, we present the detailed case study where findings are presented and discussed. Finally, we summarize our conclusions and suggest future research directions in Section 5.

## 2. Supporting Literature

### 2.1. Programme Management

Since its inception in the 1960s, project management has become an increasingly popular framework to manage business endeavours [13] and has been subjected to study in supply chains [14]. More recently, programme management has been recognised as an area of practice that is related to but different from project management [15,16]. A limited number of definitive sources on programme management specify different variations on the approach. According to Wagner [17], these include Cabinet Office [18], Project Management Institute (PMI) [19]. For example, a programme can be defined as “a collective of related projects coordinated to achieve desired benefits more effectively than when managing them as a group of individual projects” [20] or “a group of related projects managed in a coordinated way to obtain benefits and control not available from managing them individually” [21]. Individual project success is often measured by time, cost, and quality, whereas programme performance is determined by achieving a client organisation’s strategic objectives and benefits. 

Because a programme involves many projects, the need arises to group projects for technical and managerial purposes. Different criteria can be used to group projects such as project size (e.g., in monetary value or number of activities), geographical location, and technical work content. Weaver [22] proposes four dimensions to describe a programme’s characteristics: size measured in programme value, degree of technical difficulty, complexity of relationships with the stakeholders, and the degree of uncertainty. He states that the complexity of relationships is inherently indefinable and unpredictable and that the relationships within programme networks are nonlinear.

### 2.2. Programme Types and Lifecycles

Pellegrinelli et al. [23] report empirical research on how processes and techniques are applied in programme management in various contexts. Pellegrinelli and Parlington [24] argue that contextual factors attract managers’ attention and affect how they design and reshape a programme. A programme evolves with the dynamic organisational setting, which requires managers to shape both the internal and external programme context [25]. Yet, the context within which organisations deliver programmes and projects has received patchy attention in empirical studies. The lifespan and number of projects within different types of programmes vary. Pellegrinelli et al [23] states that the lifespan of a software programme is nearly two years while major construction programmes can last much longer. Financial programmes may only need to handle less than ten projects concurrently while water programmes contain thousands of projects and their sizes vary to a large extent. For industries such as the financial and service industries, the programme value may be worth less than those in capital industries, such as construction, utility, and aerospace. 

The Charted Institute of Building (CIOB) [18] states that the programme lifecycle is comprised of six stages: inception stage, initiation stage, definition stage, implementation stage, benefits review and transition stage, and closure stage. However, some authors prefer four stages: identification, definition/planning, execution, and closure [19,26,27]. The identification phase covers defining the objectives and the scope of the programme, planning and organising projects, and resource management [27,28,29]. This phase includes setting the context for defining and planning projects as part of the programme [29], so that the team members can make sense of its environment, can generate ideas, and can develop goals and courses of action [28]. The definition/planning phase focuses on refining the programme vision and objectives and on establishing the structure to facilitate project management [27,28], as well as on programme scoping and on managing risks [10].

### 2.3. Partnering and Supply Chain Integration

The multiplicity of connected projects in programme management creates the potential for copious interaction between organisations and individuals, and therefore, partnering looms as an important issue. Partnering is a broad notion including joint ventures, alliances, and framework agreements. Gadde and Dubois [30] observe that partnering in the construction industry is often limited to individual projects, but where partnering is applied, the emphasis is on suppliers providing a full solution [31]. As part of the partnering approach, relationships between a company and its suppliers can be viewed as a series of dyadic buyer–supplier relationships, e.g., between the buyer and its tier 1 suppliers. However, partnering is more than this; it is also about the supplier–supplier relationships among the individual suppliers or, as some would term it, a triadic relationship [32]. Such a perspective links easily with a social network perspective.

In the decades since 2000, supply chain integration (SCI) has come to prominence in the supply chain management (SCM) literature [33,34] and authors have identified SCI as an important element of SCM. Stevens and Johnson [35] observed “supply chain integration is the alignment, linkage and co-ordination of people, processes, information, knowledge, and strategies across the supply chain”. The influence of closer integration on improved supply chain performance has received much attention [7,8,9]. In their literature analysis, Mustafa Kamal and Irani [34] stated that, among the drivers of SCI, the second most frequently identified was “effective coordination and communication” while the third was “facilitating information sharing”, thus demonstrating the importance of communication. Stevens and Johnson [35] draw attention to the importance of communication and relationship management with partners in SCI.

According to Briscoe and Dainty [36], SCI is an elusive goal in the construction industry because of the large number of partners and their fragmentation. Cheng et al. [37] state: “Unfortunately, the construction industry is arguably the least integrated among all the major industrial sectors.” This integration deficit seems to be mirrored by the lack of reported research on SCI in this industry. For example, Mustafa and Irani [34] report that only 3.4% of papers reviewed were categorized as construction sector related.

### 2.4. Communication and Networks

In the literature, various dimensions of relationships are recognised. One of the basic aspects is communication [38,39,40]. In a classic paper, Ackoff [41] points out that, despite what managers might think, informal communication between organisational members is a necessity and not a hindrance to the formal communication required for organisations to function. 

Communication and trust are interlinked [42,43] in that communication between partners is important in building trust. One of the prevalent descriptions of trust in the literature is taken from Morgan and Hunt [44]: “when one party has confidence in an exchange partner’s reliability and integrity” (p.23). Dwyer et al [45] contend that trust emerges as one of the salient factors influencing the interaction in organisational relationships. They also point out that trust is an iterative process, during which partners trust each other to perform tasks. After a time period, partners act in the interests of the relationship and trust increases. Gulati’s findings [46,47] suggest that, as relationship formality decreases, trust grows. Laan et al. [48] observe that trust does not develop automatically, particularly in the construction industry given its history of adversarial relationships.

“Networks” as a method has gained popularity when studying relationships in a variety of fields [49]. Provan et al [49] argue that since networks can be studied from so many perspectives that “it is not always clear what organisational scholars are talking about when they use the term”. In social network theory and the related approach of social network analysis (SNA), networks refer to “a set of nodes (e.g., individuals, organisations, etc.) linked by a set of social relationships (e.g., overlapping membership, friendships) of a specified type” [50]. Similarly, Wasserman and Faust [51] state a social network “consists of a finite set of actors and relation or relations defined on them.” SNA can be used as an analytical method to map out the relational characteristics of actors in a network. Nohria and Eccles [52] stress the importance of adopting a network perspective when studying organisations because all organisations are social networks. Granovetter [53] proposes that social networks play a vital role in providing conduits through which information flows. Burt said, “people and organisations are not the source of action so much as they are the vehicles for structurally induced action” [54]. Networks can be divided into “informal networks”, that mainly depend on trust and embedded social relationships for transactional purposes [55], or “formal networks”, where communication flows reflecting authority and responsibility are governed rather than occur serendipitously [56]. Networks can also be classified as internal or external to the organisation. Much of the literature shows that (external) network resources are valuable resources that either stem from the firm’s ties to external parties [46,57] or are derived from a firm’s prior relationships. 

### 2.5. Social Networks

Gulati et al. [58] contend that the performance and behaviour of organisations can be more fully understood by examining their network of relationships. The literature describes business relationships with arm’s length relationships at one end and strategic partnerships at the other end. The literature on interorganisational relationships (IORs) and networks and collaborative forms (e.g., partnerships and alliances) is well established theoretically [46,59]. However, according to Pellegrinelli [10], the study of IORs is rarely seen within a complex network of multiple organisations engaged in a managed programme of work.

SNA was an appropriate tool to analyse relationships in this context through its ability to help understand both the static (cross-sectional) position and dynamic (longitudinal) development of networks. SNA’s capability to visualise networks using software was considered particularly useful to facilitate engagement with industrial sponsors [60]. The SNA technique uses proxy measures of interactions between individuals to identify network structure and evaluates structure through network metrics such as density. 

In fields such as supply chain management and operations management, various studies have applied SNA. SNA helps understand personal relationships or interorganisational and intraorganisational relationships which influence communication and performance through information sharing, opportunism, cooperation, and so on. Borgatti and Li [11] highlight the link between SNA and SCM. SCM literature has frequently concentrated on relational matters, through the links between supplier and buyer, supplier and customer, focal firm and multiple suppliers, and so on. SCM not only has focused on dyadic relationships but also has considered networks of relations among suppliers of suppliers and customers of customers. Carter et al. [61] point out that SCM researchers import concepts from the sociological area of SNA. SNA has also been applied in the field of project management [62,63], while in programme management, its use is very sparse. Pryke [64,65] establishes SNA as a methodology in analysing construction project relationships. He theorises that SNA provides quantitative data and accessible graphical representations of the changes in project actor’s roles and their relationships during implementation of construction projects.

## 3. Research Propositions

### 3.1. Factors Influencing the Communication Network 

Rivera et al. [66] reviewed the sociological literature that examined the processes by which ties form, persist, and dissolve in social networks. They identify three key mechanisms which they term (a) associative, (b) relational, and (c) proximity. Associative mechanisms are concerned with how the similarity of actors’ attributes affects their tie strength. Relational mechanisms draw attention to the influence of existing relationships and network positions. Proximity mechanisms focus on the social nature of the interaction in time and space. However, they do caution that, although they use these as distinct categories, these categories are intimately interwoven in the dynamics of actual social networks.

#### 3.1.1. Associative

Actors who have similar attributes are more likely to form ties. For example, in a multi-organisation team, actors who are from the same organisation are more likely to talk together than those who are from different organisations. This tendency for similar actors to form ties is termed homophily. Homophilous relationships could form between those actors who carry out technically similar work in that they may have similar education, skills, and experience that relate to this work. The above discussion leads to the following propositions for the studied context:

P1: Organisational affiliation of programme management participants affects the communication network. Affiliation to the same organisation creates stronger ties than otherwise.

P2: Affiliation of programme participants to project groups affects the communication network. Affiliation to the same group creates stronger ties than otherwise. 

#### 3.1.2. Relational

Whether actors have had prior contact or whether they are coming together for the first time will have some impact on relationships. Those actors with prior contact, whether direct or indirect, are more likely to form ties in a new network. If one of the actors in a dyad extends the offer of a relationship, then this is likely to be reciprocated. In social networks, actors cluster, i.e., they are likely to create ties with the business associates of their business associates. One of the key determinants of ties between actors is their network positions, e.g., subordinates and their superiors will need to communicate because of their roles in the network. Where actors have had prior relationships, changed circumstances can affect matters. 

P3: Seniority and industry tenure of programme participants affect the communication network favourably. 

#### 3.1.3. Proximity

The importance of proximity in enabling individuals to develop relationships is evident in several scholars’ work. In the housing industry, Duberley and Johnson [67] find an effect of proximity on the relationship between the client and the contractor. They argue that relationships of a more adversarial nature became more transactional because of closer proximity. Similarly, proximity of partners has a bearing on the degree and effectiveness of collaboration [68]. Knoben and Oerlemans [68] and Rivera, et al. describe dimensions of proximity as geographical proximity and nonspatial proximity. Knoben and Oerlemans [68] refer to proximity as “being close to something measured on a certain dimension”. In this study, proximity is specified as geographical distance between employees of different organisation working in the same location to achieve the goals of the asset management programme.

P4: Proximity of programme participants affects the communication network. Physical closeness enhances tie strength between participants.

Finally, we argue that relationships will develop as time progresses. For example, trust is expected to become stronger over time [46,47].

P5: As time elapses, communication networks will strengthen.

## 4. Methodology

This section presents the main research approaches adopted in this mixed method study. The philosophy of pragmatism underpins the research given its real-world practice-oriented nature [69].

### 4.1. Research Methods

A case study approach was adopted [70,71]. At the start of the research, effort was expended to understand the industry and case study context; this required substantial activities and time to gather information about characteristics of the industry setting and supply network configurations. The targeted case study was a UK water company’s managed programme of asset renewal and maintenance. The water company had agreed on a joint research with the university; thus, an action-learning style was appropriate given the need for the academic researchers to work closely with the programme management practitioners to gain access to the programme context and to achieve mutually beneficial objectives [72]. This approach involved academics agreeing to study objectives with practitioners and feeding back progress to practitioners for their evaluation and benefit. The mutually agreed objectives were to investigate factors influencing communication between the network of participants involved in programme management at the top of the supply chain. The study included both cross-sectional and longitudinal elements. 

Because a programme is such a complex undertaking involving many projects and a variety of stakeholders, the case study looked at the relationships between the employees of the utility company (UC), i.e., tier zero, and its contractors (CTs), i.e., tier one suppliers, at the start of the programme. The selection of CTs was often based on the bidding process, and during the investigation, interorganisational communication became the key for programme completion. The study focuses on the network of communication among participants. A range of factors were studied to ascertain their influences on the network communication in the upper level of the programme supply chain. These factors included the following:

- Organisational affiliation of individual participants (P1)
- Project grouping (i.e., how project work is allocated to groups) and the affiliation of individuals to these groups (P2)
- Characteristics of individuals including seniority and industry tenure (P3)
- Proximity of individual participants (P4)
- Elapsed time through the programme lifecycle (P5)

### 4.2. Data Collection Process

The study was designed with two main stages. At first, semi-structured interviews were carried out with senior members of the UC programme management team. These interviews helped the academic researchers to understand the research context in more depth and to establish consensus with company management on the study aims and the detailed activities. The second stage was a longitudinal analysis of the communication network between the top level of the UC programme management team and its tier one suppliers. Key data for the analysis were obtained from a questionnaire survey of key employees in the UC programme management team and in the tier one suppliers; the survey was repeated after six months to facilitate the longitudinal analysis. Snowball sampling has often been used to check the list of people and to identify further participants [51].

Social network analysis (SNA) [51,73] was selected to study the relationships among the key staff in the partnering organisations given its appropriateness to networks of substantive numbers of actors, as in this case. Collecting data on relationships between the large volume of interacting employees meant that using methods such as content or thematic analysis of interview data would be too time intensive. Similarly, using a large-scale questionnaire coupled with multivariate analysis did not seem appropriate to capture the richness of social relationships. 

The social network survey was developed to capture people’s communication activities and a measure of trust between individuals. The survey was designed to collect data via a short questionnaire electronically administered to a constrained population, i.e., a roster [73]. The roster of 110 individuals was determined in conjunction with senior managers and comprised their immediate reports within the UC and the equivalent managers and reports within the tier one contracting partners. The SNA survey was administered on two separate occasions to capture network developments; the first occasion was the fifth and sixth months of the programme, and the second was the twelfth month. The first data set (Phase 1) was collected over a seven-week period. One hundred and ten individuals were identified on the roster, and a response rate of 93% was obtained. Data gathering in phase 2 had to extend over three months because it coincided with the year-end reporting period, although this had not been realised when the timings had been agreed with senior managers. One hundred and fifteen individuals were listed on the second roster due to minor changes in personnel during this period. Despite the problems indicated above, a satisfactory response rate of 90% was obtained. A high response rate is required in SNA and was achieved by, for example, the use of a short questionnaire and targeted respondents [74].

### 4.3. The Data Collection Instrument

The survey questionnaire comprised four questions where a respondent was asked to provide information about their communication with, and views of, all other staff listed in the roster. Demographic details were also established such as the respondent’s organisation, job status, length of tenure, and their involvement in previous programme cycles. The four questions are comprised of three on informal and formal communication and one on preferred idea-sharing as a proxy for trust. Question 1 looked at general communication by asking who respondents talk to in the workplace and, thus, explored both formal and informal paths. Questions 2 and 3, on the other hand, explored more formal, directional information flows by concentrating on actors seeking information from other actors (question 2) and providing information to other actors in the group (question 3). Question 4 concentrated on trust among actors in terms of whom they prefer to share ideas with. Each respondent was asked to provide information about their relationships with all other staff listed in the roster. Respondents indicated the strength of tie using a four-point scale ranging from 0 (not at all) to 3 (a great deal). Questionnaires were sent out electronically to the key contact of each organisation or work group to ensure support from top management. These senior managers distributed questionnaires to individual respondents who were requested to return them to the researchers. 

### 4.4. Data Analysis Process

The data were analysed using UCINET [75], and NETDRAW was used to present the network data. Sociograms were used as a key tool in this process. In a sociogram, the points/nodes signify actors and the edges/ties represent the relationships of the actors with one another. Sociograms were produced for the communication networks and were analysed by factors such as organisational affiliation. Phase 1 results were presented to the senior managers in the water company’s programme team and active discussion took place between these managers and the academic researchers. To ensure confidentiality, the researchers did not reveal the names of individuals when presenting results. Analysis of phase 2 data led on to a longitudinal comparison with the two sets of cross-sectional analyses. 

### 4.5. Validity and Reliability

Various steps were taken throughout the study to ensure the credibility of emerging research findings. A quality checklist was followed as suggested by Miles and Huberman [76] for qualitative study to ensure the analysis processes and the results. The reliability and validity were assured by considering the following: the questionnaire protocol was reviewed and revised by experienced academics and practitioners; data were collected from a substantial number of programme participants; and the study’s generalizability was limited by the use of a case study linked to a particular sector and specific operations philosophy. However, such limitations are to be expected in middle range theorizing.

## 5. Case Study Details

This case study focuses on one of the UK’s largest water utility companies (UC). Project numbers and their variation in size, technical work content, and value indicate the challenging complexity of the water programme. The projects vary in technical work content since different types of capital infrastructure are dealt with, e.g., pumping stations, reservoirs, and pipeline renewal. Project values range from less than £2 million to £15 million. In addition, complexity stems from the involvement of a sizeable network of organisations, such as major contractors, subcontractors, suppliers, consultants, and regulatory institutions. Because the water programme typically engages with multiple stakeholders and is a long-term endeavour, effective interorganisational relationships between the water company and its major first tier contractors are important for programme performance. 

In delivering its capital programme, UC chose to adopt a partnering approach with its main contractors. The collaborative network studied here comprises the utility company (UC) and its main eight contractors (CTs), with the UC adopting a programme management approach to working with its CTs. UC management looks to design contracts to encourage competition between partners and to stimulate innovation. They change organisational structures frequently, particularly for each AMP cycle, as the regulator rewards companies for introducing managerial innovation as a source of “efficiency saving”. 

UC managers believe the company’s success relies heavily on partnership with its tier 1 contractors. A CT is typically formed by a partnership between a design company and a civil construction company, an arrangement with what could be termed tier 2 suppliers. In successive cycles of asset management programmes, UC has employed a different mix of tier 1 contractors. These changes stem from Ofwat encouraging a “shake up” in each AMP.

**Figure 1:**
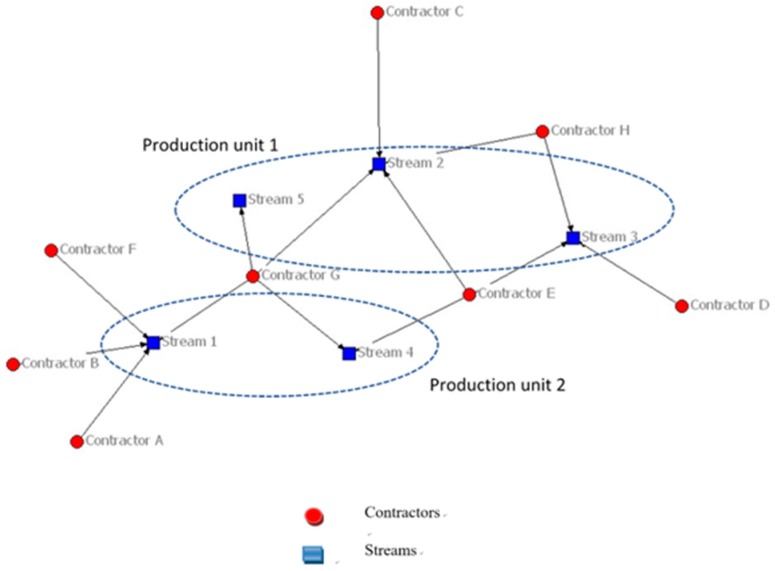
Contractor–Stream Relationships.

Two major changes were instituted at the start of the studied programme: (1) key personnel from all nine organisations were co-located in a newly acquired multistorey office block to form a newly created Asset Delivery Unit (ADU) and (2) project work was organised into five streams containing projects of a similar technical nature, under two higher level “production units”. The project grouping determines the work allocation to stream members and establishes key relationships between employees. Each stream has a UC manager who was also in charge of lower-level work allocations by batch and project. Contractors operate in different numbers of streams, ranging from one to four streams (see Figure 1); however, individuals are often restricted to working on one stream or sometimes two. Some contractors have prior relationships with the UC from previous AMPs. Collaboration between contractors is expected across both production units and all five streams as part of the performance criteria established as a response to incentives from the regulatory body. Production Unit 1 comprises streams 2, 3, and 5, while Production Unit 2 is made up of streams 1 and 4. A stream level analysis, amongst others, was applied in the research to investigate the connections among the nine organisations (UC and the eight CTs). 

In Figure 1, each contractor is anonymised by using a capital letter (A–H), while each stream is represented using a number (1–5). The combination of eight contractors with five streams of work adds complexity to the analysis of relationships. For instance, contractor G is assigned to streams 1, 2, 4, and 5 while contractors A, B, C, D, and F work for only one stream. Each stream is independent from each other but related in the programme. Furthermore, prior association with UC varies to a large degree. Contractors B and D are new to working with UC on a programme for this cycle, whereas contractors G and E had collaborative relationships with UC for 15 years through three programme cycles of association. 

## 6. Findings

First, the cross-sectional findings for phase 1 are presented, and then, by presenting the findings for phase 2, a longitudinal view is presented. 

### 6.1. Phase 1

#### 6.1.1. Communication

**Figure 2:**
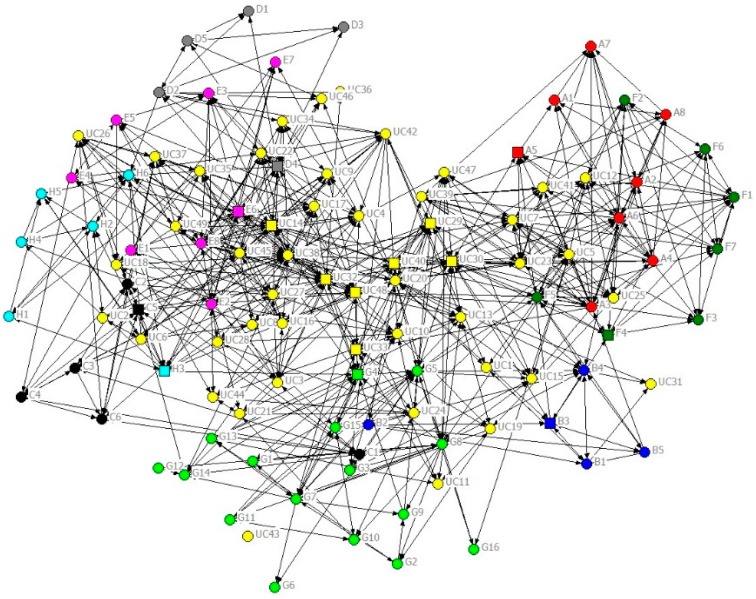
Phase 1 sociogram for general communication (strength of tie = 3 and organisation and key contact attribute display).

A sociogram showing the general communication network between roster members is shown in Figure 2. Each node represents an individual, and an arc connecting two individuals shows they are connected by a strong tie (rated 3). The individual’s organisational affiliation is designated by the node colour (see Table 1 and Table 2), and the status of the individual is designated by the shape of the node (key contacts are square-shaped, and other staff are circles).

The UCINET software places those nodes with the highest number of connecting ties toward the centre of the network. That is, those individuals rated as involved with the most frequent communication are placed at the centre, while those with the least frequent communication are placed at the periphery. The utility company employees (yellow nodes) are clearly located more towards the centre of the sociogram than the contractor employees who are grouped around the periphery. The correlation of node positioning with the organisation type (UC—tier zero and CTs—tier one) is to be expected as a reflection of the power imbalance between the tiers. It is also noticeable that individuals from the same organisation tend to be placed near one another, thus reflecting that an individual is more likely to communicate with someone in their own organisation. 

The extent of communication in the whole network can be measured by network density. A density of 100% would represent a network where every node was connected to every other node; in that case, everyone strongly communicates with everyone else. The density of this communication network was 53%, a high density, since this means that 53% of all possible ties (at strength 3) were present. This percentage means that actors on average talk to just over half of the other individuals listed in the roster. Therefore, even when newly established, i.e., only six months old, this network was quite dense (as were the other networks). This high density could be partly explained by relationships existing between some individuals prior to the programme establishment. Demographic data from the questionnaire indicated that some individuals had served the water industry for a long time even when tenure within their current organisation appeared to be very short. This indicated that some individuals were potentially well-connected with others even if their organisation was “new” to this programme. Within the sociograms, various individuals with long industry service appeared to be located towards the network centre because of prior relationships with UC personnel during previous cycles. The UC senior managers and the contractor’s key managers were also located more towards the centre, reflecting the formal status of their roles.

The sociograms for more formal communication, i.e., questions 2 and 3 (not shown here because of space constraints), were less dense (35% and 31%), indicating the more constrained nature of formal communication. However, the network densities were slightly different with the seeking information network having a slightly higher density than information providing.

In general, the structures of the two networks were similar, reflecting the reciprocal nature of the communication, i.e., one person’s information seeking is the other person’s information provision. However, detailed examination of the networks did reveal one particular individual in one of the contractors who claimed to provide a great deal of information to other contractors but received very little information from them, thus illustrating how human nature can impact data accuracy. Notwithstanding such detail, the structures of the formal communication networks were like that for general communication in that the UC employees were more centrally located in the networks and employees of individual contractors were clustered close to each other. 

#### 6.1.2. Trust

**Figure 3:**
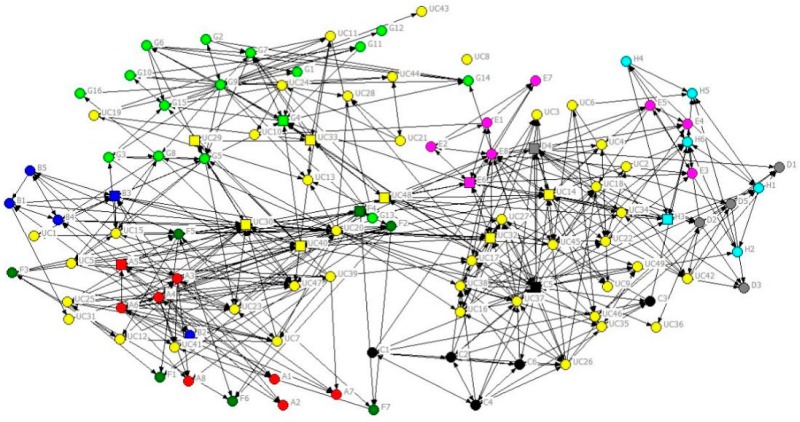
Phase 1 sociogram for preferred idea sharing (strength of tie = 3). Same organisation and key contact attribute display in Figure 2.

Figure 3 shows the network for the trust question. This sociogram illustrates various aspects. First, the UC staff (yellow nodes) tends to be well-distributed among those for the CTs, indicating their mutual preferences for ideas sharing (trust). Second, the key managers of the CTs (square nodes) are also positioned more toward the centre of the network compared to other staff within their organisations, thus demonstrating higher trust for higher status individuals.

**Figure 4:**
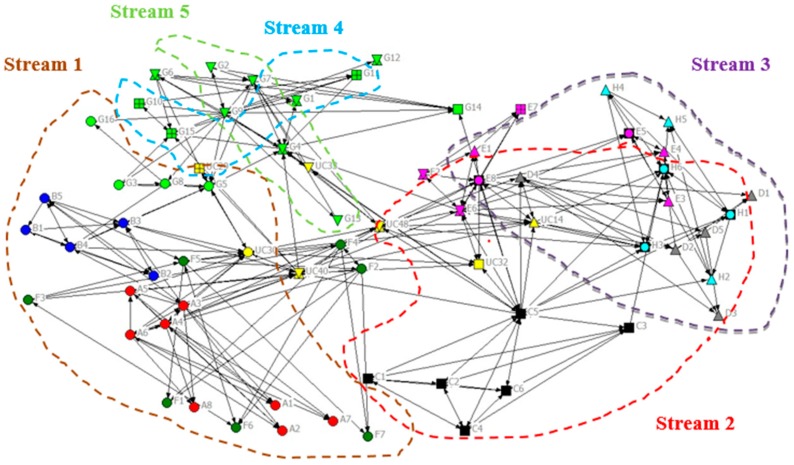
Phase 1 sociogram for preferred idea sharing (strength of tie = 3 and organisation and stream attribute display).

To more clearly discern the impact of project grouping on trust, Figure 4 excludes all UC managers except the seven most senior managers of the two high-level production unit groups and five subordinate streams whilst retains all contractor staff. To see more clearly the project groupings, coloured boundaries enclosing all the nodes (individual) belonging to the stream are added to Figure 4 (see Table 3). The situation is not entirely clear cut since some individuals have roles in more than one stream; however, the network splits into two “halves” with each half comprising a production unit. Production Unit 1, comprising streams 2, 3, and 5, appears to form a substantially “tighter” sub-network than Production Unit 2 (streams 1 and 4). Similar structures can be observed in the communication networks.

#### 6.1.3. Proximity

Key personnel from all nine organisations (UC and eight contractors) were co-located in a new multistorey office block. Because of the numbers involved, the personnel from UC and the contractors occupied four contiguous floors of the office block. The general allocation was that the top two floors contained staff from the streams of one production unit while the bottom two floors were dedicated to staff from the streams belonging to the other production unit. Streams 2 and 3 seem well interconnected in the sociogram for Figure 4 (both Unit 1, one floor apart). Stream 4 seems highly integrated with stream 5, despite them being located on floors 4 and 3, respectively, and belonging to different production units. However, the influence of proximity is not that easy to clearly discern given that the staff numbers render it difficult to place related individuals in one physical location. 

### 6.2. Phase 2 Compared with Phase 1

**Figure 5:**
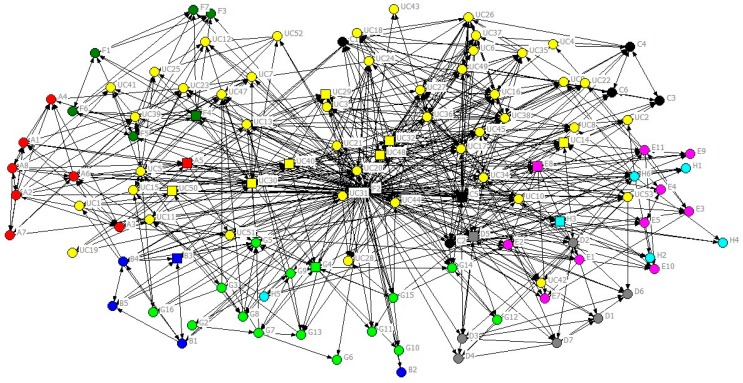
Phase 2 sociogram for preferred idea sharing (strength of tie = 3; organisation and seniority attribute display). Same organisation and key contact attribute display in Figure 2.

To examine the evolution of communication and trust networks over time, the sociograms for phase 2 were constructed and compared with the relevant ones for phase 1. For example, Figure 5 shows the sociogram for trust at phase 2 which should be compared with Figure 3, which shows the equivalent network for phase 1. In Figure 3, there was a very clear partition of Production Unit 1 and Unit 2, which was much less pronounced in the phase 2 sociogram. Another feature of the phase 2 trust sociogram was the migration of UC’s nodes inwards from their phase 1 positions, which were located more toward the periphery. This movement could indicate that UC was taking the lead in idea generation and sharing or that contractors were letting them do so and becoming more “hands-off”.

We surmise here that significant increases in measures of formal communication and trust from phase 1 to phase 2 can be attributed to accumulated work activity and that participants become more assured about their formal work relationships as the programme develops and becomes bedded in. Conversely, general communication was already at a high level, which was achieved early in the programme cycle and, given this communication’s informality, its frequency would not be enhanced by the development of increased expertise in more formal areas of activity. 

## 7. Discussion

In general, the results support the propositions made in the literature review. The sociograms clearly demonstrate how individuals affiliated with the same organisation were clustered together (P1). Similarly, the organisation of projects into production units and streams based on technical work content influences the communication networks (P2). The influence of seniority and industry tenure on relationships is also evident in the sociograms in that individuals with more seniority and more tenure are more centrally located in the network (P3). The influence of physical proximity is not that easy to discern given that the high numbers of staff rendered it difficult for UC to concentrate all related individuals close together. A further problem in ascertaining the influence of proximity on communication and trust is that stream affiliation and physical location are confounded variables (P4). The significant increases between phase 1 and phase 2 in the network densities for formal communication and trust demonstrate the impact of elapsed time (P5).

One of the major aspects dealt with in this study is the influence that the method of grouping programmes into work packages has on the programme relationships. Previous programmes delivered by the water company illustrate some of the variety in which work packages can be grouped, i.e., projects were grouped according to different criteria. For example, in a previous programme, CTs were allocated to contractor groups located in different geographical regions of UC’s territory. Projects below a certain monetary value were awarded automatically to regional groups if they fell within their specific region; for higher value projects, any contractor, irrespective of their regional affiliation, could tender competitively. In the following programme, contractors were named as “partners” of the UC with some changes to the supplier entities and an attempt was made at partial co-location, with a number of UC managers assigned to each of the contractors’ regional offices. Some CTs have collaborated with the UC across several programmes, and one may observe that, in the studied programme, the CTs with the longest relationship with UC in asset delivery tended to be awarded the greatest number of stream memberships. Clearly, various factors would have informed the award of contracts, but the connection between allocation of stream memberships and prior relationships is nonetheless striking. While in general, maintaining and developing client–contractor relationships in successive programmes can be recommended as a good strategy, this only applies up to a point. Injections of new partners may mitigate against complacency in established relationships.

Proximity is an important factor in facilitating communication networks. Notwithstanding the contribution made nowadays by Information and Communication Technology (ICT), physical location is still important. However, the case study shows that, in a large programme, sheer size can mitigate against achieving close physical proximity between managerial participants and, therefore, may dilute the achievement of benefits. However, physical proximity is still believed to be an important factor in the programme because the similarity of work content provides a stronger platform for need-driven communication between actors [77,78].

Efficiency incentives promoted by the regulator have influenced behaviours within the industry, sometimes in counterintuitive ways. For example, water companies are tempted to pursue radical change in supply networks and business models for each programme simply to capture innovation-related rewards rather than to obtain resource efficiencies. Each water programme contains considerable investment in physical infrastructure. With increasing pressure from regulation, the relationships between industry stakeholders have become more complex and demanding for water companies, which inevitably impacts how they integrate with partners. 

Although programme phases are described as different sections of the lifecycle, certain activities overlap phases and connect them together; for example, the results of the identification phase and the definition/planning phase will typically affect the performances of the execution and closure phases. Similarly, although relationship development occurs throughout the lifecycle, the early programme phases are where the initial formation of relationships is expected to take place. In this initial phase, the organisation that leads or “owns” the programme appoints their partners in the supply chain, e.g., tier 1 suppliers. This is where initial relationships are formed by the establishment of communication networks. The exploration of relationship evolution and development along the programme lifecycle is one of the important themes attracting attention in the network, relationships, and interorganisational partnership literature.

Here, the early network benefitted from a reasonably dense communication pattern for “preferred ideas sharing” i.e., “trust”, as demonstrated in Figure 2. Co-location undoubtedly played a part in establishing this; however, the nature of work organisation posed a much stronger impact upon network structure than anticipated. The fact that this presented itself at the production unit level as opposed to the stream level is of worthy note and likely to reflect in some way upon the individual production unit managers and mobilization of their respective stream managers. Network density clearly increased between phases 1 and 2; Figure 5 illustrates the strong UC communication together with dominant individual communicators at the centre of the network. One can reflect upon how the production unit pattern within the original “preferred communication” network has lessened, suggesting that a more collaborative and solution-oriented approach has evolved over time. Within the field of programme management research, this is a positive illustration of increased collaboration at multiple levels (production unit and stream) of work organisation over time.

The use of SNA in this study was novel both as a research method and as a mechanism for informing management. The method appears not to be used a lot in programme management research but is used more so in project management. SNA’s use in programme practice also appears to be low. The researchers found that sociograms were useful for communicating with management. However, presenting results to and discussing them with industry participants also better informed the researchers’ interpretations of the sociograms. The discussions also helped achieve agreement between the practitioners and academics on the goals and conduct future study activities. However, a downside to this use of SNA was that the promise of anonymity to survey participants came under pressure during feedback sessions with managers. Whilst the researchers resisted this pressure and anonymity as far as possible, for certain analyses, it was deemed better to gain agreement from participants to their waive anonymity in order to present more meaningful sociograms; fortunately, all the individuals to whom this applied agreed.

Much of the research on programme management focuses on the programme manager and on the required characteristics and competences of that individual. However, teamwork is emphasized in business and management theory and the large size of many programmes can be argued to mitigate against overemphasis on the one individual. A novel and valuable aspect of this study is that it goes some way to redressing the balance by concentrating on a wide social group of managers and on their integration. Moreover, as argued in the introduction, the study’s context is a novel one with its location in the privatized but monopolized water sector in UK. This novel context and the use of the case study method leads to caution over generalizing to other sectors in line with middle range theorizing. 

## 8. Conclusions and Future Research

### 8.1. Study Outline

The paper presents empirical research on supply chain partnership communication in the context of a water company’s asset management programme, across early stages of the programme lifecycle. The research involved close collaboration between researchers and the water company management, and the research adopts both qualitative and quantitative methods. The study used social network analysis (SNA) to study how relationships between key staff in the partnering organisations are influenced by various factors including organisational affiliations, work allocation, and proximity. The early qualitative interviews complemented the later quantitative analysis by enabling joint understanding by researchers and company management of the real-life problems present in the programme and established consensus on the implementation of the social network analysis questionnaire survey. The longitudinal study with repeated surveys provides insight into how the water company collaborates with CTs in the asset management supply networks and shows how networks change over time. 

### 8.2. Contribution to Theory and Implications

In the programme we studied, the water company initiated project grouping (i.e., stream) based on technical work content as one of the organisational changes at the early stage of the programme. The study shows that the structures of communication networks are strongly influenced by the individuals’ affiliations to project groups (streams). Project grouping is a key element of programme management, particularly in those programmes comprising large number of projects. A stream level of analysis was, therefore, an appropriate method to better understand relationships, in general, and communication, specifically, between contractors and the water company. The study highlights the complexity of relationships that arises from the decisions taken in the managerial structures to deal with work allocation. In this case, the complexity was exacerbated by contractors serving different types and numbers of streams ranging from only one stream to four different streams. Other factors influencing the communication network include factors such as the individual’s affiliation to organisations, their seniority and industry tenure, and their proximity to one another. The study also showed how the communication network strengthened over time, in that the network densities increased. Additionally, the study contributes to the academic literature by applying theory on social networks and relationships to programme management in the regulated water industry. The context presented here is particularly novel and complex and raises interesting challenges for network performance and development.

### 8.3. Contribution to Practice and Implications

The UK water industry has faced and continues to face ongoing challenges of climate change, household growth, and higher customer expectations while under stringent regulations. In such a challenging context, water company managers need to be as effective as possible in managing their companies and their relations with partners. Apart from contributing to the literature, this research assisted water company management in better understanding the nature of relationships in their programme and the factors influencing relationships. Additionally, the study introduced programme managers to SNA as an effective management tool worthy of adoption more widely within the water company and the programme management environment in general. 

The findings have shown stream membership, proximity, and other factors’ impact upon relations in the water sector. The research could contribute to wider practice, both to programmes in the wider UK utility sector and in regulated industries worldwide and to programme management practice in general.

### 8.4. Limitations and Future Research

Resource constraints attached to the study meant that the research had to focus on a single utility company and its next tier down from suppliers. The five-year length of the programme exceeded the one-year allotted to the empirical study and, thus, constrained the study to part of the programme lifecycle. However, concentration on the early part of the lifecycle was felt to be more useful given that this is where relationships are established. 

Given the focus on a single case study, wider studies of more utility programmes within the industry would be useful. The case study approach enables the researcher to study a programme, process, individuals, or organisations in depth, but it has its limitations. The generalisation from this study to wider contexts may be somewhat hampered by using a case study method and the novelty of the context, i.e., a privatized, monopolised utility environment. Given the novelty of the context, opportunities exist to widen out research to look at other programme types in other contexts. Future research could look at more extensive periods and cover the whole of the programme lifecycle. This research’s exploratory nature leads to suggesting that more in-depth explanatory research could be suitable to pursue in future. 

## Figures and Tables

**Table 1:** Colors of Organisations in Figure 2.

Organisation	Colour		
A	Red	F	Dark Green
B	Blue	G	Light Green
C	Black	H	Light Blue
D	Grey	UC	Yellow
E	Pink		

**Table 2:** Status of the individual in Figure 2.

Key Contact Attribute	
□	Key Contacts in Contractors, UC Stream Managers and UC Production Unit Managers
O	Other Staff

**Table 3:** Attribute of individuals.

Individuals’ Stream Membership Attribute			
Shape	Stream	Shape	Stream
Circle	O Stream 1	Down triangle	∇ Stream 5
Square	□ Stream 2	Circle-in-box	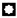 Streams 2&3
Up triangle	△ Stream 3	Egg timer	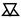 Other *
Box	⊞ Stream 4		

* Other refers to a combination of two or three different streams.
